# Two Sisters with Kallmann Syndrome, Gonadal Dysgenesis, and Multiple Neuromuscular and Endocrine Disorders: Report of Two Cases with Description of an Unusual Association

**DOI:** 10.1007/s43032-022-00897-z

**Published:** 2022-02-23

**Authors:** Marta Camacho, Camil Castelo-Branco

**Affiliations:** Clinic Institute of Gynecology, Obstetrics and Neonatology, Faculty of Medicine, University of Barcelona, Hospital Clinic-Institut d´Investigacions Biomèdiques August Pi I Sunyer (IDIBAPS), Barcelona, Spain

**Keywords:** Kallmann syndrome, Ovarian dysgenesis, Mitochondrial encephalomyopathy

## Abstract

Kallmann syndrome (KS) is an uncommon genetic disorder characterized by isolated congenital hypogonadotropic hypogonadism (CHH) and anosmia/hyposmia. KS originates from abnormal embryonic migration of olfactory axons and gonadotropin-releasing hormone (GnRH)-synthesizing neurons. It can be challenging to diagnose due to its heterogeneous clinical presentation and genes implied. Herein, we report a rare phenotype of KS in two sisters accompanied by a variety of nonreproductive disorders such as hypoparathyroidism, hypercortisolism, atrophy of the cerebellum, intellectual disability, and remarkably, ovarian dysgenesis. Additionally, both subjects present muscle weakness, exercise intolerance, marked hypotonia and seizures, being suspected, although not fully confirmed, mitochondrial encephalomyopathy. These cases illustrate the heterogeneous clinical presentation and the diagnostic difficulties often found in patients suffering from this condition. These clinical features have never been described before as associated with KS; therefore, we decided to report this novel KS phenotype.

## Introduction

CHH is primarily caused by a GnRH deficiency, leading to low levels of gonadotrophins and sex steroids. In approximately 50% of the cases, CHH patients also present a defective sense of smell, which is then known as KS. Historically, KS and the normosomic CHH variant have been well differentiated. Nevertheless, recent studies suggest that from a clinical standpoint, these two entities should be considered two different expressions of the same pathology as they share common features and genetic etiopathogenesis [1, 2]. They are proposed to be considered the same complex disorder and so-called isolated gonadotropin-releasing hormone deficiency [3].

In association with CHH, a wide spectrum of nonreproductive features and developmental abnormalities have been reported, although its prevalence is not well established yet. The most commonly described abnormalities include microphallus, cryptorchidism, synkinesis, hearing loss, renal anomalies, cleft lip/palate, and dental agenesis [1, 2]. Ovarian dysgenesis is a congenital developmental disorder of the ovary that causes hypergonadotropic ovarian failure. It is therefore not likely to be found in patients with KS. Nevertheless, this finding has exceptionally been described in the literature in association with KS [4].

The diagnosis could be a formidable challenge due to its heterogeneous presentation. If it is recognized during the early stages, an appropriate approach and management of the disease can be made to minimize the long-term effects of hypogonadism [5].

In recent years, genetic diagnosis has changed as new genes and inheritance patterns have been identified. Also, additional phenotypic features newly described could be used for the prioritization of genetic screening [8].

Herein a report of two sisters affected by this condition is presented, highlighting phenotypic variability, diagnostic complexity, the importance of tailored treatment, and the need for future research.

## Case Report

A highly exceptional case of KS in two sisters without substantial family background prompted this report. Permission from the family and patients was obtained prior to the publication of this article, as well as approval of the ethics committee (HCB/2020/1392).

**Fig. 1:**
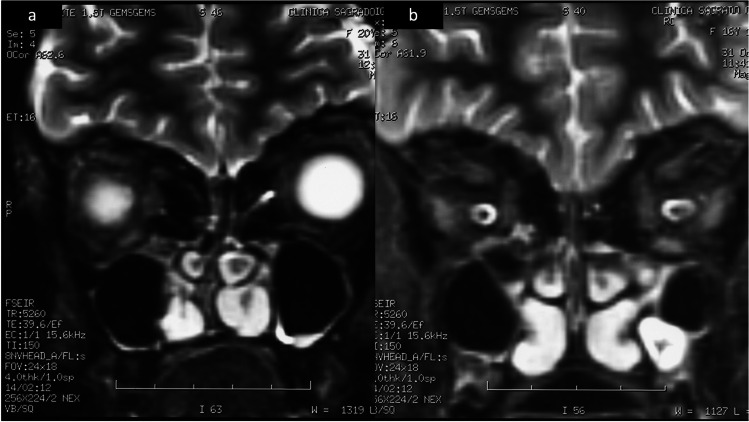
Brain MRI revealed aplasia in the olfactory bulbs and tract in both patients (case 1 (**a**) and 2 (**b**))

Case 1: The first patient is a female of 34 years old who was diagnosed with hypogonadotropic hypogonadism at the age of 16, and the suspicion of KS was confirmed at the age of 27. She presents the following nonreproductive features: hypoparathyroidism, hypercortisolism, intellectual disability, and interatrial communication. Due to her marked hypotonia, exercise intolerance, developmental delay, muscle weakness, and episodes of seizures, at the age of 20, a muscle biopsy was performed, and she was also diagnosed with mitochondrial encephalomyopathy due to the presence of COX-negative fibers with a deficit of complex I of the respiratory chain. Furthermore, because of her condition, at the age of 23, she was diagnosed with osteopenia. From an early age, she demonstrated neurodevelopmental disorder and intellectual disability. Multiple studies during her infancy and childhood were performed. A karyotype found no abnormalities (46 XX); sequencing of the mitochondrial DNA found no disease causing; and CAT and MRI of the brain described cerebellum atrophy, a microadenoma in the pituitary gland, and calcifications at the basal ganglia. At the age of 16, due to the absence of secondary sexual characters and the lack of growth spurt, further studies were made. Analysis showed indetectable sexual steroids and gonadotropins (< 0.10 UI/L) being compatible with congenital hypogonadotropic hypogonadism. At this point, ultrasonography and computerized tomography of the pelvis were performed, showing a rudimentary uterus and the presence of streak gonads. Laparoscopy with ovarian biopsies was performed, confirming gonadal dysgenesis: neither follicles nor germ cells were found in the biopsy. A growth hormone stimulation test revealed a partial deficit of the growth hormone (GH). Consequently, she was treated with GH replacement therapy that lasted for 6 months. After ending the GH replacement therapy, she began with estrogen therapy for 6 months. Since then, she has been under hormone replacement therapy with combined estrogen and progesterone. At a later stage, Kallmann syndrome was suspected as hyposmia was detected. A genetic test to detect KAL1 (ANOS1) and KAL2 mutations was performed, but both were negative. A comparative genomic hybridization was performed, resulting in a loss of heterozygosity in chromosome 13. Another brain MRI was performed and confirmed previously undetected aplasia in the olfactory bulbs (Fig. 1a).

Case 2: The second patient, a sister of case 1, presented a developmental course, diagnosis process, and diagnostic studies almost analogous to those previously described. She is a female of 29 years old who was diagnosed with CHH at the age of 14, and the diagnosis of KS was established at the age of 22. She presents the following nonreproductive features: hypoparathyroidism, high cortisol levels, intellectual disability, mitral valve prolapse, and subclinical hypothyroidism. She also presents muscle weakness, exercise intolerance, and marked hypotonia. Her muscle biopsy was considered normal, but a diagnosis of mitochondrial encephalomyopathy was made, assuming it was a false negative.

She was diagnosed with spina bifida at birth and also presented neurodevelopmental disorders during early life, resembling patient 1. The same studies carried out for patient 1 were performed during her early life: karyotype and mitochondrial DNA sequencing, both with no abnormalities. Computerized tomography and MRI of the brain also described cerebellum atrophy and calcifications at the basal ganglia. Like her sister, she exhibited linear growth but not growth spurt during adolescence. At the age of 14, she was detected to have a deficiency in growth hormone and CHH, after which she began GH treatment. A year later, at 15, estrogens were added to the GH therapy. Since 16 until now, she has been receiving hormone therapy with estrogen and progesterone. Just like occurred to patient 1, image studies suggested rudimentary uterus and streak ovaries; thus, a laparoscopy and an ovarian biopsy were performed, and gonadal dysgenesis was confirmed. Further on, anosmia was also detected, and KS was suspected. In an analogous manner to that of her sister, no mutations were found in KAL1 and KAL2, but it was confirmed on her a loss of heterozygosity of chromosome 13. Furthermore, at the age of 17, she was diagnosed with osteopenia. Brain MRI revealed a mega cisterna magna, atrophy of cerebellar vermis, small pituitary gland, and aplasia in the olfactory bulbs and tract (Fig. 1b).

## Discussion

We herein present an unusual case of KS in two sisters associated with other rare conditions. To the best of our knowledge, KS has never been associated with mitochondrial encephalomyopathy, and its association with gonadal dysgenesis has been exceptionally described.

Although a hereditary condition is suspected, the genetics in patients 1 and 2 remain unclear. It seems unlikely to be sporadic cases, and regarding inheritance patterns, we could reject an X chromosome-linked recessive pattern, therefore not likely to present a mutation in the KAL1 (ANOS1) gene. The comparative genomic hybridization performed in both patients did not report which part of chromosome 13 was involved in the loss of heterozygosity, hindering the possibility of finding a candidate gene involved in the etiology. Conversely, chromosome 13 has not been related to any genetic forms of HH or KS specifically [9]. The authors made an effort to review genes located in chromosome 13 to hypostasized a possible correlation and found that 13q33-q34 microdeletions are related to developmental delay and/or intellectual disability, increased risk of epilepsy, hypotonia, cardiac defects, and additional anatomic anomalies [10], suggesting the possibility that an aberration in genes located in this area could contribute to the etiology of patients 1 and 2 conditions. Regarding these facts, the current assumption is that there is an oligogenic mechanism underlying the condition of these two patients, but we cannot discard an autosomal dominant inheritance pattern with incomplete penetrance or even an autosomal recessive pattern. In order to shed light on the inheritance pattern of this unique phenotype, authors suggest deeply studying chromosome 13 in these patients (more specific 13q33-q34 region) and performing a genetic analysis of their parents.

Although genetics remain to be fully elucidated in KS, recent research has led to the identification of more than 60 genes that might be implicated in approximately 50% of the cases [1, 6, 9]. CHH/KS could be presented as either sporadic or familial cases. Additionally, several inheritance patterns have been described (autosomal recessive and dominant and X-linked recessive) with different penetrance. In this context, the novel paradigm is to consider the hypothesis of oligogenic and gene-environment interaction [7].

A proper genotype–phenotype correlation might aid in addressing genetic testing and to personalizing genetic counseling and screening [8]. Some specific mutations have implications for clinical features, such as synkinesis (KAL1), dental agenesis (FGF8/FGFR1), hearing loss (CHD7), and finger bones abnormalities (FGF8/FGFR1) [8, 9]. Our patients do not present synkinesis, renal or dental agenesis, or cleft/lip palate. Patient 2 does have hearing loss. Regarding other nonreproductive features: patient 2 underwent surgery because of congenital flat feet and was diagnosed with congenital interauricular communication, which has been described as associated with KS [8]. Intellectual disability has previously been described in males that present a contiguous gene syndrome, a large deletion in Xp22.3 that results in X-linked ichthyosis and KS [11]. As patients 1 and 2 have no deletions in the X chromosome and do not present ichthyosis, this mutation could be discarded. Intellectual disability has also been related to mutations in gene CHD7, responsible for CHARGE syndrome (coloboma, heart anomalies, choanal atresia, intellectual disability, genital/urinary defects, and hearing loss) [6]. Intellectual disability and CHH normosomic have been associated with mutations in the gene DMXL2 [3]. Jain et al. [12] report a case of a patient with the CHD7 mutation associated with hypoparathyroidism and basal ganglia calcifications at the CT scan (both clinical features of our patients) but without hypogonadism or defective sense of smell. In patients with KS, aplasia or hypoplasia of the olfactory bulb can be identified on brain MRI. However, these traits do not always correlate with the sense of smell.

Mitochondrial myopathies are a group of heterogeneous disorders caused by an impairment in the oxidative phosphorylation pathways of the respiratory chain. The diagnosis is usually suspected by a detailed history and physical examination and can be confirmed by molecular genetic studies, muscle biopsy, and additional tests such as neuroimaging and electromyography [13, 14]. In the light of highly variable and overlapping phenotypes, it is challenging for clinicians to diagnose it accurately [15]. The association between KS and mitochondrial encephalomyopathy has not been described yet. Nevertheless, mitochondrial encephalomyopathy, intellectual disability, infantile hypotonia, and cardiomyopathy in patients affected by a mutation in gene CHKB are associated [16]. Although in both patients the history, physical exam, and neuroimaging are suggestive of mitochondrial myopathy, we consider a shortcoming of the diagnosis in patient 2 the fact that her biopsy did not test positive for the basic histochemical stains and reactions suggestive of mitochondrial disease.

The most exceptional finding in these patients is the fact that gonadal dysgenesis was demonstrated at the ovarian biopsies of both. To our knowledge, there is only one case report in the literature in which ovarian dysgenesis has been manifested concurrently with KS [4]. Gonadal dysgenesis is caused due to abnormal germ cell migration and/or organization in the genital ridge [15]. This disorder of sex development results in hypergonadotropic ovarian failure, as sex steroid secretion is deficient, resulting in elevated FSH levels. As we previously described, KS is not caused by an embryologic abnormality of the gonads but of the GnRH synthesizing neurons, therefore, causing a hypogonadotropic hypogonadism condition.

It is also noteworthy that some studies have described impairment of smell in patients with hypergonadotropic hypogonadism, more specifically, in patients with Turner syndrome [16]. This common feature also suggests a possible association in the genes involved in the etiopathogenesis of some hyper and hypogonadotropic hypogonadisms.

Appropriate management is crucial to diminish health consequences [17]. The lack of sexual development importantly influences the sexual functioning and quality of life of women affected by CHH [18]. In order to induce sexual development or fertility, adequate treatment and counseling should be addressed. Patients 1 and 2 received hormonal replacement treatment in order to develop secondary sexual characteristics but not to induce fertility due to their condition.

These two cases highlight the genetic and phenotypic heterogeneity of KS and spotlights that there is still a broad array of unanswered questions. We contemplate that further research is needed to determine the molecular basis correlating CHH, intellectual disability, gonadal dysgenesis, and mitochondrial encephalomyopathy underlying these two cases as it has not been reported in the literature before. Specifically, we highly encourage to deeply study possible aberrations in 13q33-q34 in these patients and their parents in order to eventually shed light on its inheritance pattern.

## Data Availability

All data is available if requested.
